# Verification of dynamic and segmental IMRT delivery by dynamic log file analysis

**DOI:** 10.1120/jacmp.v3i2.2578

**Published:** 2002-03-01

**Authors:** Dale W. Litzenberg, Jean M. Moran, Benedick A. Fraass

**Affiliations:** ^1^ Department of Radiation Oncology University of Michigan Medical Center 1500 East Medical Center Drive Ann Arbor Michigan 48109‐0010

**Keywords:** IMRT, quality assurance, sequence verification, dynamic log file

## Abstract

A program has been developed to evaluate the delivered fluence of step‐and‐shoot segmental and sliding window dynamic multileaf collimator (MLC) fields. To automate these checks, a number of tools have been developed using data available from the dynamic log files that can be created each time a dynamic delivery occurs. Experiments were performed with a Varian 2100EX with a 120 leaf MLC equipped with dynamic capabilities. A dynamic leaf sequence is delivered and measured with film or an amorphous silicon imager. After delivery, the dynamic log file is written by the accelerator control system. The file reports the expected and actual position for each leaf and the dose fraction every 0.055 seconds. Leaf trajectories are calculated from this data and expected and actual fluence images are created from the difference of opposing leaf trajectories. These images can be compared with the expected delivery, measurements, and calculations of fluence. Tools have been developed to investigate other aspects of the delivery, such as specific leaf errors, beam hold‐off flags sent by the control system to the MLC, and gap widths. This program is part of a semi‐automated quality assurance (QA) system for pretreatment fluence verification and daily treatment verification of dynamic multileaf collimation (DMLC) delivery.

PACS number(s): 87.53.–j, 87.52.–g

## INTRODUCTION

Quality assurance of IMRT delivery techniques such as segmented multileaf collimation (SMLC) (step‐and‐shoot) and dynamic multileaf collimation (DMLC) (sliding window) is critical to ensure accurate delivery of optimized treatment plans. The proper implementation of IMRT, using dynamic multileaf collimation (MLC) techniques, requires a thorough understanding of leaf motion during delivery. Most often the effects of leaf motion are inferred from dose deviations on film or an electronic portal imaging device.^1^ ^–^ ^5^ Likewise, the dosimetic effects of leaf position uncertainty may be determined from variations in ionization measurements.^6^ These techniques provide dosimetric information but do not provide detailed information for diagnosing delivery problems. Other verification techniques that calculate the fluence distribution from the MLC leaf trajectory file have the advantage of not requiring sequence delivery, but only consider anticipated delivery constraints; not necessarily constraints such as the tolerance setting, dose rate, programmed monitor units (MU), or system delay time.^7^ These techniques require that the behavior of all clinically relevant parameters and system characteristics be programmed into the verification software. Behavior that is not anticipated or understood could otherwise pass verification. More specific evaluation of the control system and MLC function can be done using the information contained in the dynamic log files, or “DynaLog Files” in the case of Varian MLC's.^8^ ^–^ ^11^ These files contain leaf position and dose fraction information recorded roughly every 0.055 seconds. This information can be used as part of the overall system QA to evaluate the function of different parts of an IMRT system.^9^ ^,^ ^12^

To aid in the evaluation of DMLC delivery, software was developed to read in the DynaLog files and provide analysis and evaluation tools to assess routine QA and clinical leaf sequences, and provide additional information that cannot be determined from dosimetric measurements.^13^ Using the information provided in the file, the software calculates kinetic, dosimetric, and statistical properties of the delivered sequence. The purpose of this paper is to describe the analysis program and demonstrate its functionality for commissioning and routine QA of IMRT techniques.

## MATERIALS AND METHODS

### Application environment

This software is written in an analysis package called IGOR Pro^a^ (Interactive Graphics Oriented Research) that includes a visual programming language, very similar to C, and provides calls to its analysis and customizable graphics capabilities. IGOR also provides extensive capabilities for automated analysis, image processing and analysis, and full features for input and output of ascii and binary files, among other capabilities.

### DynaLog file description

The software was developed to test sliding window DMLC and SMLC delivery of IMRT on a Varian Clinac 2100EX accelerator equipped with a 120 leaf MLC.^b^ The vendor's control system creates a record summarizing, approximately every 0.055 seconds, the machine status (beam on or off), the dose fraction, expected and actual leaf positions, MLC beam hold‐off flag and beam‐on flag at each interval, and various other parameters such as the user‐selected tolerance. This record may be written to a file (DynlogA.txt and DynlogB.txt) on the control system computer after each DMLC field delivery. A complete file description may be found elsewhere.^c^ These files can be transferred to another computer for detailed analysis of the operation of the DMLC function after each IMRT field is delivered.

### Automation

One of the primary goals is to automate, to the extent possible, the acquisition, analysis, comparison, and storage of data for verification and refinement of DMLC and IMRT treatments. In this paper we will focus on the analysis of the DynaLog files. At this time, the DynaLog files are created, named, and stored manually. The dose and dose rate information (not stored in the files) is entered into the program to scale the dosimetric results appropriately. The user then selects the DynaLog files for analysis. After reading the DynaLog files, all calculations are immediately performed.

### Quantities calculated

The quantities calculated and displayed for evaluation may be grouped into four categories: position versus time related quantities, and their derivatives; dose scaled quantities versus position (created by inverting and interpolating the dose fraction and position data); differences between the desired and actual values for the previous quantities, and statistical data calculated from these quantities. The first three categories are very useful for pinpointing specific difficulties in delivering a sequence. The statistical quantities are useful for quantifying overall delivery characteristics.

Statistical quantities are calculated (i) for all leaves, (ii) for leaves that modulate fluence (i.e., move across the field during the delivery), and (iii) for leaves that modulate fluence but only when the beam is on. Statistics under conditions (i) are easy to calculate but skew results by including data from uninvolved leaves. This is only useful when looking for consistency in delivering the same sequence many times. Statistics calculated under conditions (ii) tend to give more meaningful absolute results for dynamic delivery techniques such as the “sliding window” method where the beam is expected to be on during the entire sequence. Statistics calculated under conditions (iii) are much more useful for looking at segmental sequences when the beam is shut off between segments. In this delivery technique it is clinically irrelevant where the leaves are or how they move while the beam is off.

A sample of some of the calculated quantities is shown in Table I. These are global quantities that may be used to characterize various aspects of a sequence delivery. Table II shows a sample of some of the displays and quantities that are calculated for individual leaves or leaf pairs. Quantities that are provided in the file and are not calculated, such as beam hold‐offs versus time/dose fraction, are not included in the table but may also be displayed.

**Table I:** Global quantities and displays for delivery evaluation.

Quantity or Display
Average and RMS position deviation under conditions (i), (ii), and (iii)
Number of MLC beam hold‐offs
Percent time beam was held off by MLC
Time beam held off
Total treatment time
Image display of expected fluence vs position and/or time
Image display of actual fluence vs position and/or time
Image display of fluence difference vs position and/or time
Image display of leaf position deviations vs time for bank A and B
Image display of leaf position tolerance faults vs time for bank A and B

**Table II:** Leaf specific quantities and displays

Quantities and Displays
Calculate position deviation vs time for each leaf
Calculate RMS position deviation for each leaf
Calculate expected and actual velocity vs time of each leaf
Calculate expected and actual acceleration vs time of each leaf
Calculate and display expected gap vs time for each leaf pair
Calculate and display actual gap vs time for each leaf pair
Calculate and display gap deviation vs time for each leaf pair
Calculate average and RMS gap
Calculate and display expected and delivered leaf trajectories
Calculate and display expected and delivered dose profiles for each leaf pair
Calculate and display dose discrepancy profiles for each leaf pair
MU scaling of leaf trajectory and dose related quantities and graphics
Histograms of position deviations for each leaf
Histogram of leaf position deviations when beam is on and/or not held off
Histograms of actual velocities for each leaf
Histograms of actual acceleration for each leaf
Histogram of expected and actual velocity when beam is on and/or not held off
Histograms of actual velocities for each leaf
Histogram expected and actual gap and gap deviation for each leaf pair
Histogram gap deviations for conditions (i), (ii), and (iii)
Graphical comparison of all expected and actual quantities for each leaf
Tables summarizing each leaf's position deviations
Tables summarizing each leaf's expected and actual velocities
Tables summarizing each leaf's expected and actual accelerations
Tables summarizing each leaf's expected and actual gap sizes and gap deviation statistics.

### Visualization and display of results

The manner in which the results are displayed has a strong impact on the user's ability to evaluate the performance of the control system and MLC in delivering a sequence and in troubleshooting problems. Results are displayed in four ways. Tables and graphs are typically used for statistical quantities. A single quantity may be graphed for all leaves in a bank on one graph (histograms of position deviation, for example). This type of display is very useful for QA in spotting failing motors. Other quantities are compiled into grayscale or color images showing that quantity versus leaf number and time or position to provide quick visual verification or identification of difficulties. And finally, additional graphs are used to display different quantities and state variables versus time or position for one leaf or leaf pair. This simultaneous display of various types of information on one graph is an important feature for immediate comparison of many quantities to aid in evaluation of the delivery. This is very important in trouble shooting and aids in understanding cause and effect.

**Figure 1:**
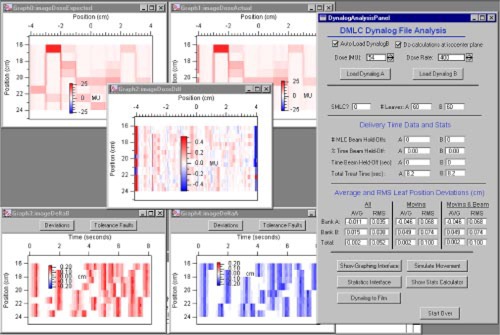
(Color) The primary DynaLog File Analysis interface and automatically displayed result images. The fluence distributions shown at the top are reconstructed from a dynamically delivered sequence. The difference of the expected and actual fluence distributions is shown in the middle, while leaf position deviations are shown in the bottom two images.

The primary user interface is shown on the right side of Fig. 1. After entering the total MU delivered and the dose rate, the user selects the DynaLog files for analysis. The DynaLog files are read and all calculations are immediately performed. Statistics on the number of MLC beam hold‐offs are displayed in the interface window along with statistical results on position deviations for each bank of leaves. Images are automatically displayed that show reconstructions of the desired fluence distributions (top left) and the actual fluence distribution (top right) (including transmission). The difference between the desired and actual distributions is displayed in the center image.

The bottom images show the magnitude of leaf deviations versus time. The “Tolerance Faults” button is used on either graph to redefine the color scale to show only deviations that exceed the user‐defined tolerance value. Typically, the initial analysis and display takes less than three seconds and depends on the size of the DynaLog file. These results and images provide a quick visual verification that the desired distribution was delivered and which leaves may be involved in delivery discrepancies.

The five buttons at the bottom of the primary interface, shown in Fig. 1, provide access to other interfaces for displaying results. These include an interface for displaying time and position related quantities for each leaf, statistical results, graphically simulating the desired and actual leaf motions,^d^ and utilities for comparing the reconstructed distributions to measurements made with digitized film, an amorphous silicon imager, or dose calculation maps based on optimized fluence grids.

**Figure 2:**
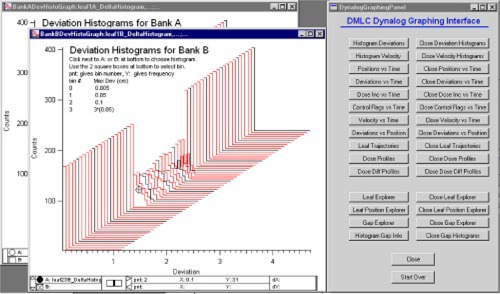
(Color) The Graphing Interface and the displays generated when the “Histogram Deviations” button is clicked. These types of displays show data for each leaf in each bank. Values may be obtained using the cursors at the bottom of the graph.

Figure 2 shows the Graphing Interface and the displays generated when the “Histogram Deviations” button is clicked. Many of the other buttons produce similar displays, showing data for each leaf in a bank. (Values for each leaf are easily obtained by dragging a cursor to the desired location as shown in the image.) Other buttons provide access to delivery status parameters that vary with time such as the beam‐on flag and the MLC beam hold‐off flag.

**Figure 3:**
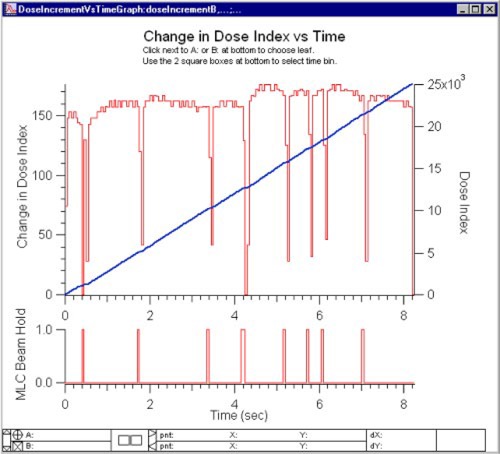
(Color) This figure shows the cumulative dose fraction index in the top graph (blue line). From this cumulative index, the incremental change (top graph, red line) in the index is determined to evaluate variations in the dose rate during delivery. The MLC beam hold‐off flag is displayed at the bottom for easy comparison.

The cumulative dose fraction is recorded in the DynaLog Files every 0.055 seconds, indicating the fraction of the total treatment given up to that point. Regardless of the number of MU delivered, the fraction is scaled from 0 to 25 000 in the file (instead of from 0 to 1). Figure 3 shows the cumulative dose fraction index in blue on the top graph (note that this is a dimensionless quantity). From this cumulative index, the incremental change (shown in red) of the index is determined to evaluate variations in the dose rate during delivery. On the same graph, the MLC beam hold‐off logical is displayed at the bottom. This allows easy visual determination of how the dose rate varies when the MLC exerts a beam hold‐off when a leaf goes out of tolerance.

**Figure 4:**
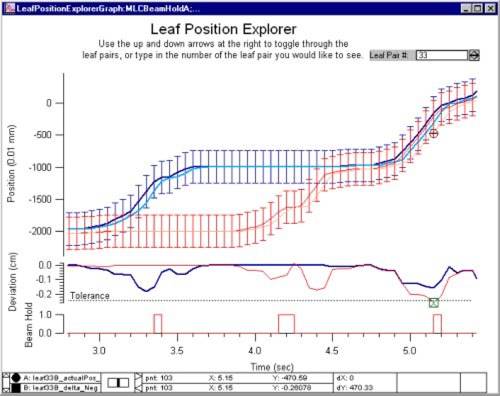
(Color) The “Leaf Position Explorer” displays the desired and actual positions vs time for each leaf in a pair, the deviations from the desired position, the user‐set tolerance, and the MLC beam hold‐off flag.

The bottom group of buttons in the “Graphics Interface” (Fig. 2) provide access to various “Explorers.” These are displays that show many results for one leaf, or leaf pair, simultaneously, along with global status parameters, such as the beam‐on flag and the beam hold‐off flag. The “Leaf Position Explorer” is shown in Fig. 4. The control at the top of the graph allows the user to toggle through or type in the leaf of interest. The dark blue and red lines show the desired leaf position, for both leaves in a pair, and error bars show the user set tolerance. The lighter blue and red lines show the actual leaf positions as reported in the file. To easily identify tolerance faults, the tolerance value and deviations from the desired position are shown below the position data. The MLC beam hold‐off flag is shown at the bottom. During the time interval shown in this example, leaf pair 33 went out of tolerance 5.15 sec into the sequence. Three other tolerance violations occurred, in other leaves, during the time interval shown as indicated by the beam hold‐off flag.

**Figure 5:**
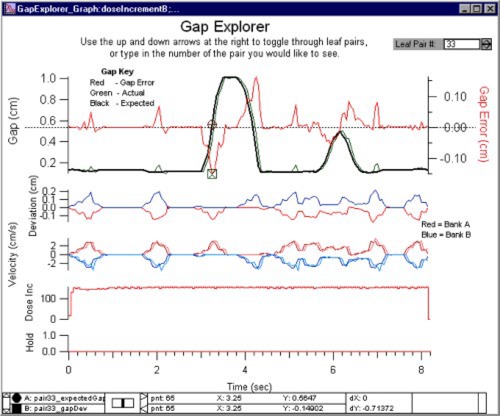
(Color) The “Gap Explorer” display shows the desired and actual gap for a given leaf pair and the error in the gap. It also shows the deviation in position for each leaf, the desired and actual velocities of each leaf in the pair, the change in the dose fraction index, and the MLC beam hold‐off flag.

Figure 5 shows the “Gap Explorer.” This display shows the desired and actual gap for a given leaf pair and the error in the gap. It also shows the deviation in position for each leaf, the desired and actual velocities of each leaf in the pair, the change in the dose fraction index, and the MLC beam hold‐off flag. In addition to the data display, the desired and actual gap data are scanned for negative values indicating leaf collisions. The number of collisions per leaf pair is printed in a summary table along with the number of times each leaf went out of tolerance. (This summary also contains statistical and logistical information about the delivery and analysis of the DynaLog files.) As with the other Explorers, the desired leaf pair is displayed using the control at the top of the graph.

Similarly the “Leaf Explorer” displays many quantities for a single leaf. These include the leaf position and deviation, velocity, acceleration, MLC beam holds, and cumulative and incremental dose index versus time. The expected and actual values for each quantity are both displayed for comparison where applicable. As with the other Explorers, a control at the top of the graph allows the user to toggle through or type in the leaf of interest.

Other tools have been developed for image processing, analysis and comparison. This will allow us to investigate the potential use of an amorphous silicon imager in automated DMLC delivery verification and QA. The “DynaLog to Image” button presents an interface that allows images to be imported, processed and compared to each other. Images containing dosimetric or fluence information (film, amorphous silicon imager,^13^ or dose calculations based on desired fluence maps) can be imported and converted to dose, if appropriate, using measured calibration curves. The program has features to align, scale, and normalized the images among other features. Different images can then be selected for comparison. For example, dose calculations at a plane from the planning system may be compared to film or amorphous silicon imager measurements made in phantoms.

**Figure 6:**
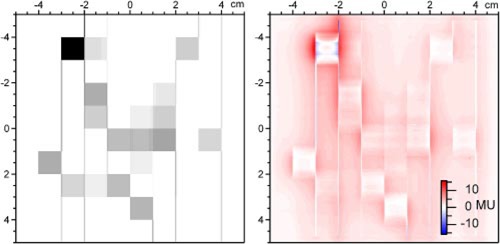
(Color) The figure on the left shows a desired distribution that was reconstructed from the DynaLog File data. The figure on the right shows the difference between an a‐Si imager measurement and the desired distribution.

One possible technique for a relative pretreatment consistency check that will be investigated using these tools is the comparison of the desired distribution, reconstructed from the DynaLog files, with amorphous silicon imager measurements made in air. For example, Fig. 6 shows the desired fluence distribution (calculated from the DynaLog File) and it's subtraction from an a‐Si imager measurement in air at 100 SSD of the delivered sequence. Strictly speaking, neither quantity is fluence or dose. The reconstructed distributions from the DynaLog files do not currently include small‐field output factor corrections, off‐axis fluence corrections, or scatter corrections; and no dose calculation is performed based on the reconstructed distribution. Nonetheless, if the comparison of these quantities, or quantities derived from them, show that the delivery will go as planned, it may prove to be a useful relative verification tool.

### Leaf motion simulation

The expected and actual leaf motion of a delivery can be graphically simulated while showing the dose fraction (much like the Varian Shaper application). This is useful for problem solving and visual verification and recognition of the sequence delivered. This interface also allows the user to step forward or backward through the expected segment shapes or through the actual measured shapes. It is also possible to overlay or flag locations where leaves go out of tolerance or collide during the motion simulation.

## DISCUSSION

It is important to note that this software tool does not obviate the necessity of routine MLC QA. Routine QA is required to ensure the MLC is operating properly and that the data in the DynaLog files is accurate. Guidelines for routine MLC QA have been previously reported.^14^ The validity of the data in the DynaLog files is dependent on the accurate readout of the leaf positions. Semi‐weekly radiographic tests may be used to identify position encoder drift. Routine QA sequences can be run and quickly analyzed to evaluate MLC performance each morning. A simple sequence that delivers a uniform field using all the leaves can be used to spot motors with deteriorating performance, for example.

Another application of this software is the daily pretreatment verification of clinical sequences. The pretreatment test sequence could potentially be imaged with an amorphous silicon imager and compared with the distribution reconstructed from the DynaLog file for pretreatment consistency checks. Once a particular sequence has been thoroughly tested and approved for clinical use, statistical values can be stored as a standard reference for daily pretreatment verification. In time, this may allow dosimetric verifications to be phased out if a strong correlation is found between measured distributions and those reconstructed from the DynaLog files. This would save time on sequence verification for the physicist. In addition, such data could allow evaluation of DMLC delivery over the course of treatment and provide feedback to actively refine patient treatments.

## SUMMARY AND CONCLUSIONS

The DynaLog files contain a significant amount of data that can be used in many ways to enhance a clinical program using dynamic delivery techniques, such as sliding window DMLC or segmental techniques to implement IMRT These uses range from evaluating simple QA sequences for routine monitoring of MLC performance to trouble shooting MLC delivery problems, to independent clinical sequence verification. Many of these checks may be automated to significantly reduce the amount of time needed by the physics staff. In the long term, automated verification systems may be possible that can track MLC performance and make dose modeling and treatment refinement possible.

## ACKNOWLEDGMENTS

We would like to thank Wayne Keranen of Varian Medical Systems for providing the DynaLog File format. Communication with Varian was greatly facilitated by Dr. Dan McShan and Dr. Marc Kessler. Support for this work was provided in part by NIH Grant No. P01‐CA59827.

## Footnotes

a WaveMetrics, Inc., Lake Oswego, OR.

b Varian Medical Systems, Palo Alto, CA.

c DynaLog File Viewer User Guide, Varian Medical Systems, P/N 10007000.

d This feature is much like Varian's Shaper application that uses the DMLC files to graphically simulate the expected leaf motion.
